# Prevalence and risk factors associated with cat parasites in Italy: a multicenter study

**DOI:** 10.1186/s13071-021-04981-2

**Published:** 2021-09-15

**Authors:** Marco Genchi, Alice Vismarra, Stefania Zanet, Simone Morelli, Roberta Galuppi, Giuseppe Cringoli, Riccardo Lia, Manuela Diaferia, Antonio Frangipane di Regalbono, Giulia Venegoni, Fabrizio Solari Basano, Antonio Varcasia, Stefania Perrucci, Vincenzo Musella, Emanuele Brianti, Alessia Gazzonis, Michele Drigo, Liliana Colombo, Laura Kramer

**Affiliations:** 1 Dipartimento Di Scienze Medico-Veterinarie, Università Di Parma, via del Taglio, 10, 43126 Parma, Italy; 2 Dipartimento Di Scienze Veterinarie, Università Di Torino, L.Go Braccini, 2, 10095 Grugliasco, TO Italy; 3 Facoltà Di Medicina Veterinaria, Università Degli Studi Di Teramo, 64100 Teramo, Località Piano d’Accio Italy; 4 Dipartimento Di Scienze Mediche Veterinarie, Università Di Bologna, Via Tolara di Sopra, 50, Ozzano Emilia, 40064 Bologna, Italy; 5 Dipartimento Di Medicina Veterinaria E Produzioni Animali, Università Di Napoli Federico II, Via Federico Delpino 1, 80137 Naples, Italy; 6 Dipartimento Di Medicina Veterinaria, Università Degli Studi Di Bari, Valenzano, 70010 Bari, Italy; 7 Dipartimento Di Medicina Veterinaria, Università Degli Studi Di Perugia, Via San Costanzo 4, 06126 Perugia, Italy; 8 Dipartimento Di Medicina Animale, Produzioni E Salute, Università Degli Studi Di Padova, Viale dell’Università, 16, 35020 Legnaro, PD Italy; 9 Arcoblu S.R.L, Via Alessandro Milesi 5, 20133 Milan, Italy; 10 Dipartimento Di Medicina Veterinaria, Università Degli Studi Di Sassari, Via Vienna 2, 07100 Sassari, Italy; 11 Dipartimento Di Scienze Veterinarie, Università Degli Studi Di Pisa, Viale delle Piagge 2, 56124 Pisa, Italy; 12 Dipartimento Di Scienze Della Salute, Università Di Catanzaro Magna Graecia, 88100 Catanzaro, Italy; 13 Dipartimento Di Scienze Veterinarie, Università Degli Studi Di Messina, 98168 Messina, Italy; 14 Dipartimento Di Medicina Veterinaria, Università Degli Studi Di Milano, via dell’Università 6, 26900 Lodi, Italy; 15 Dipartimento Di Medicina Animale, Produzioni E Salute, Università Degli Studi Di Padova, Viale dell’Università, 16, 35020 Legnaro, PD Italy; 16 MSD Animal Health, Via Fratelli Cervi, 20090 Segrate, MI Italy

**Keywords:** Cat, Parasites, Italy, Prevalence, Zoonosis, Risk factors

## Abstract

**Background:**

Parasites that infect cats include protozoa, helminths and arthropods, many of which are transmissible to humans. Effective control relies on a good knowledge of parasite distribution and the risk factors for infection. The present study was aimed at evaluating the prevalence of major feline parasites in Italy and the risk factors associated with their occurrence.

**Methods:**

Over a 12-month study period, feces, hair and ectoparasites from naturally infected cats from feral colonies, shelters and private households were analyzed at 13 study centers across Italy. Samples from these cats (*n* = 987) were analyzed at all centers using the same diagnostic methods. Prevalence values and risk factors were evaluated statistically for the identification of predictors of risk.

**Results:**

The overall prevalence of gastro-intestinal and broncho-pulmonary (BP) nematodes was 35.9% (354/987). *Toxocara cati* was the most prevalent species (253/987; 25.6%), followed by Ancylostomatidae (98/987; 9.9%). Among BP nematodes, *Aelurostrongylus abstrusus* was the most common (76/987; 7.7%). Approximately 35.7% (352/987) of the study population was infested by ectoparasites, of which the most common were fleas (29.4%, 290/987), followed by ear mites *Otodectes cynotis* (9.8%, 97/987). Predictors of risk for parasite infection included age, a predominantly or exclusively outdoor lifestyle, geographic area and lack of antiparasitic treatment.

**Conclusions:**

Both ecto- and endoparasites are still common in cats throughout Italy, many of them being of zoonotic concern and vectors of pathogens to humans. Given the presence of parasites throughout the entire study period, year-round treatment should be considered. Furthermore, data confirm the need to protect the human–animal bond using proper endo- and ectoparasiticides to reduce the risk of human infection, in application of the One-Health concept.

**Graphical abstract:**

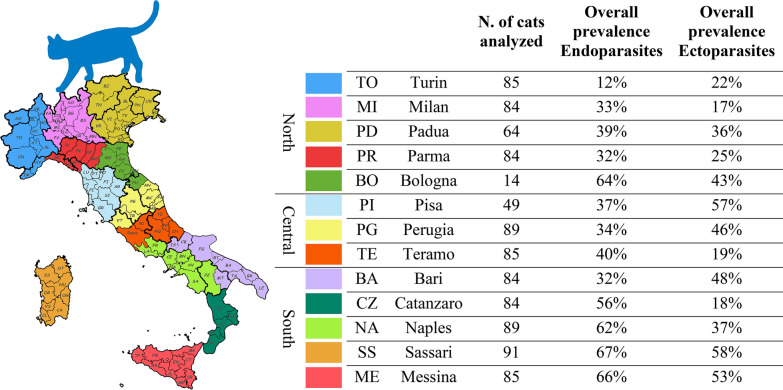

**Supplementary Information:**

The online version contains supplementary material available at 10.1186/s13071-021-04981-2.

## Background

Numerous parasites infect domestic cats. Among these, helminths of the gastro–intestinal (GI) tract and respiratory system can cause severe disease if parasite loads are heavy, while different arthropods can cause skin disease and allergy. Subclinical infection is of equal concern given the zoonotic nature of several feline helminths and the capacity of fleas and ticks to transmit pathogens to cats, other animals and humans. Recent multicenter studies in both Europe and Italy have been carried out to define the current status of endo- and ectoparasite infections of cats [1–4]. The results of these studies indicate that infections are widespread and depend on various risk factors, including lifestyle, geographical area and frequency of antiparasitic treatment. Most studies on prevalence, distribution and risk factors for feline parasites in Italy have been carried out in the central and southern areas of the country [5, 6].

Multicenter studies provide useful information on the distribution and risk of parasite infection. However, few apply the same, standard diagnostic protocols at each center, thus compromising comparability of the results. Indeed, it has been reported that different copromicroscopic techniques have differing sensitivity, specificity and accuracy for the diagnosis of GI and broncho–pulmonary (BP) nematodes.

Seasonality of parasite infection in cats has been evaluated mostly in retrospective, longitudinal studies [12, 13]. It is possible that sampling and analysis over a fixed period of time may provide more useful information on the current effects of season on parasite prevalence.

The aims of the present multicenter study were therefore to: (i) determine the current prevalence of feline endo- and ectoparasites throughout Italy through the recruitment of cats from all regions; (ii) evaluate seasonal trends by recruiting a set number of cats each month consecutively over a 12-month period; (iii) use standardized diagnostic methods in order to eliminate variables associated with differences in test sensitivities/specificities/accuracy; (iv) identify those factors that significantly increase the risk of infection.

## Methods

### Animals and study period

The study period was from July 2019 to September 2020. Thirteen university study centers participated, and each center had a recruitment target of seven cats per month for 12 months, for a total of 84 cats per center. Each cat could only be included in the study once, with no more than two cats from the same home/shelter/colony sampled. Randomization was based on the target number of seven cats: once the target number was reached, enrollment ceased even if there were other cats that met the inclusion criteria.

### Enrollment and sample collection

Inclusion criteria included: outdoor access; no antiparasitic treatment (endo and/or ecto) in the 3 months prior to enrollment; and signed informed consent. Exclusion criteria included: no outdoor access; and antiparasitic treatment (endo and/or ecto) in the 3 months prior to enrollment.

Each study center was supplied with tubes (15 ml) pre-filled with 80% ethanol; flea combs and zip-lock bags; one Mini-FLOTAC kit Fill-FLOTAC [14] containing four Mini-FLOTAC and four Fill-FLOTAC devices (200 tests); and instructions for material collection, conservation and analyses.

At enrollment, general information, inclusion criteria, clinical observations, frequency of antiparasitic treatments in the previous 12 months and eventual signs of ectoparasitic infestation were recorded, and an online data collection sheet was filled in (Additional file 1: Text S1). Any observed ticks, lice and nits were collected (mites were collected by scrapings or ear wax collection while nits, lice and ticks were simply removed with tweezers) and stored in the provided 15-ml tubes containing 80% ethanol. Each cat was combed with a flea comb for 5 min, and collected material was stored in the zip-lock plastic bag at 4 °C. Owners were asked to submit at least 7 g of fresh feces, which were collected, examined for the eventual presence of proglottids and stored at 4 °C.

### Laboratory analyses

All collected material was analyzed at the university laboratory of each participating center.

Material collected with flea combs was examined under a stereomicroscope and the presence of flea/flea debris recorded. Feces were examined for the presence of proglottids and identified according to Soulsby et al. [15].

Mini-FLOTAC copromicroscopic examination was carried out on 2 g of feces in 18 ml of NaCl floating solution (specific gravity: 1.200) according to the protocol described in Cringoli et al. [7]. Minimum/maximum number of eggs/oocysts/cysts per gram of feces (EPG/OPG/CPG) were calculated. The Baermann test was carried out on 5 g of feces and the feces examined approximately 12 h later, according to Bowman et al. [16]. Larvae were identified according to Varcasia et al. [17] and Brianti et al. [18].

Study center reference personnel were asked to register with the Castor® EDC® database [19], which was managed by the study monitor. Each reference personnel had her/his own login credentials. Results were transcribed into the database, preferably within several days of receipt and analyses.

### Statistical analyses

Chi-square tests were carried out to evaluate the association between positivity for at least one endo-/ectoparasite infection, *Toxocara cati*, Ancylostomatidae, *Aelurostrongylus abstrusus*, fleas and *Otodectes cynotis*, as well as for the following categorical variables: sex, geographical area (North, Central, South), lifestyle (exclusive outdoor/predominantly outdoor/predominantly indoor), provenance (privately owned, shelter, colony), age (< 1 year, 1–5 years, > 5 years) and antiparasitic treatment in the previous year (yes/no).

Relationships between infection and the variables (“predictors”) were analyzed by multivariable regression analysis applying a stepwise backward elimination method (IBM SPSS Statistics for Macintosh, version 27.0; IBM Corp., Armonk, NY, USA.). Statistical significance was set at α = 0.05.

## Results

### Study population

Severe restrictions on movement/activity were put into place to contain the SARS-CoV-2 pandemic (March 2020 “lockdown”), which resulted in a decrease in/lack of cat enrollment at many participating centers from March 2020 to May 2020, leading to an extension of the study for a further 2 months. A total of 987 cats were enrolled.

Cats were evenly distributed in terms of sex. Approximately half the study population (47.3%) was within the age range of 1–5 years, with the remaining cats evenly distributed between < 1 year (28.1%) and > 5 years of age (24.6%). Over 40% of enrolled cats were from southern Italy and 69.2% were privately owned cats. Over 75.8% of the study population lived predominantly outdoor/exclusively outdoor (Table 1).

**Table 1:** Characteristics of the study population

Characteristics	Values, *n* (%)	Characteristics	Values, *n* (%)
**Sex**		Number of ectoparasiticide treatments done in the last year	
Male	492 (49.8)	0	655 (66.4)
Female	495 (50.2)	1	192 (19.5)
**Status**		2	59 (6.0)
Non-sterilized	438 (44.4)	3	38 (3.9)
Sterlized	549 (55.6)	4	24 (2.4)
**Provenance**		5	7 (0.7)
Shelter	29 (2.9)	6	11 (1.1)
Colony	275 (27.9)	7	0 (0.0)
Privately owned	683 (69.2)	8	1 (0.1)
**Lifestyle**		Number of endoparasiticide treatments done in the last year	
Predominantly indoor	239 (24.2)	0	752 (76.2)
Predominatly outdoor	423 (42.9)	1	159 (16.1)
Exclusively outdoor	325 (32.9)	2	47 (4.8)
**Geographical area in ltaly**		3	14 (1.4)
North	331 (33.5)	4	10 (1.0)
Central	223 (22.6)	5	1 (0.1)
South	433 (43.9)	6	3 (0.3)
**Age (years)**		7	0 (0.0)
< 1	277 (28.1)	8	1 (0.1)
1–5	467 (47.3)		
> 5	243 (24.6)		

Most cats, including privately owned animals, had not received antiparasitic treatment in the year before enrollment. Overall, 539 of the 987 (54.6%) cats in the study were infected with one or more parasites. Of these 987 infected animals, 13.7% (*n* = 135) had at least two endoparasites and 7.4% (73) had at least two arthropod infestations.

### Endoparasites

**Fig. 1:**
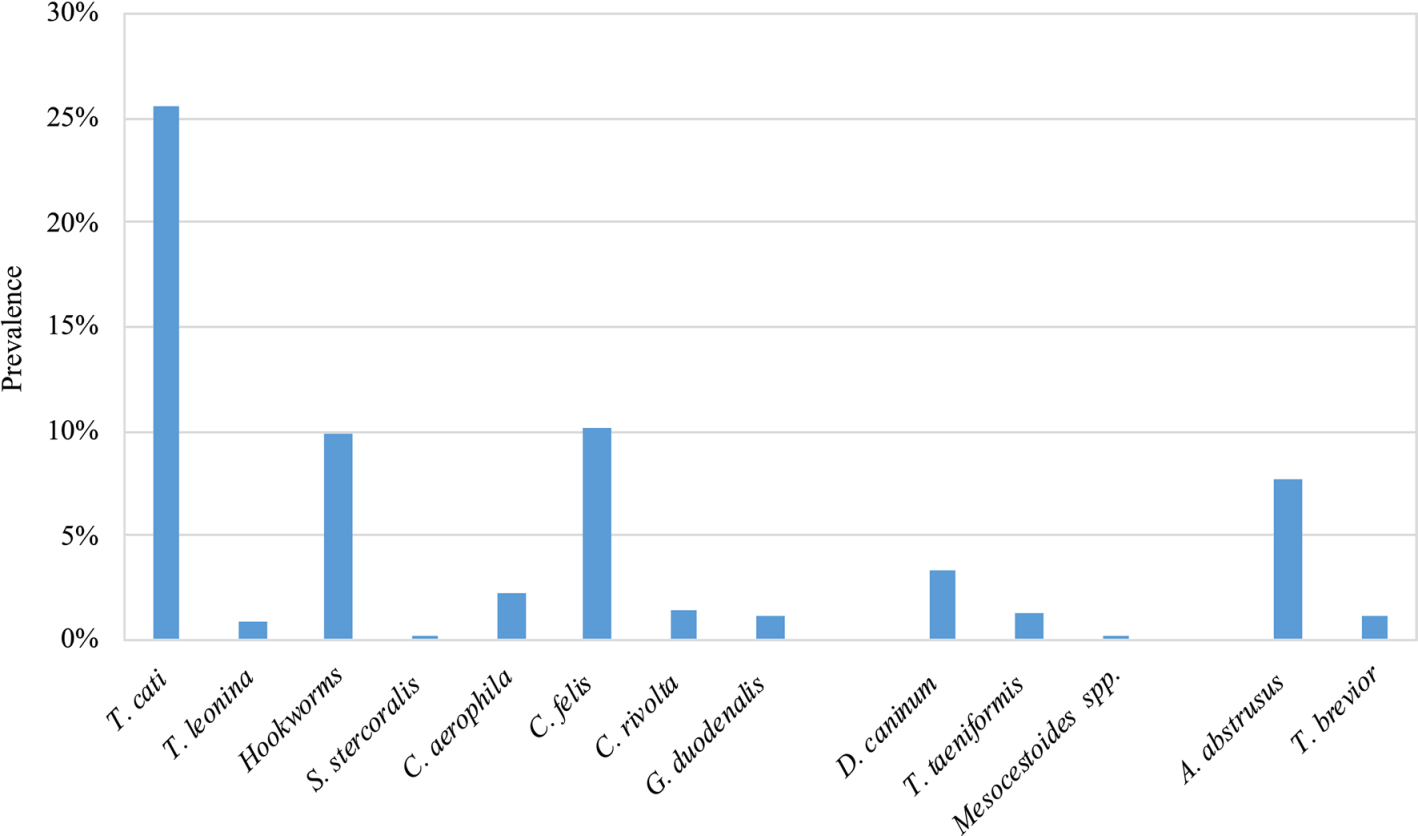
Overall prevalence of gastro-intestinal and broncho-pulmonary endoparasites

The overall prevalence of GI and BP nematodes was 35.9% (354/987). *Toxocara cati* was the most prevalent species (253/987; 25.6%), followed by Ancylostomatidae (98/987; 9.9%). Among the BP nematodes, *Aelurostrong abstrusus* was the most common (76/987; 7.7%), while *Capillaria aerophila* (*Eucoleus aerophilus*; 22/987, 2.2%) and *Troglostrongylus brevior* (12/987, 1.2%) were much less frequently found (Fig. 1; Table 2). Among the GI protozoa identified, *Cystoisospora felis* was the most common (101/987; 10.2%) while all other species were uncommon (Fig. 1; Table 2). Based on macroscopic examination of proglottids [15], members of class Cestoda were rarely found (*Dipylidium caninum* 3.3%, *Taenia taeniaeformis* 1.3%, *Mesocestoides* spp. 0.1%) (Fig. 1; Table 2).

**Table 2:** Number of positive cats and prevalence (%) of endo- and ectoparasites reported from the 13 centers (3 geographical areas in Italy) participating in the study

	Northern area					Central area			Southern area					Total,* n *(%)
Study centers:	TO	MI	PD	PR	BO	PI	PG	TE	BA	CZ	NA	SS	ME	
Gastrointestinal parasites														
Number of cats	85	84	64	84	14	49	89	85	84	84	89	91	85	987
*Toxocara cati*	8 (9.4)	21 (25.0)	19 (29.7)	12 (14.3)	8 (57.1)	14 (28.6)	11 (12.4)	16 (18.8)	15 (17.9)	37 (44.0)	29 (32.6)	28 (30.8)	35 (41.2)	253 (25.6)
*Toxascaris* *leonina*	4 (4.7)	0 (0.0)	0 (0.0)	1 (1.2)	0 (0.0)	0 (0.0)	0 (0.0)	0 (0.0)	1 (1.2)	2 (2.4)	0 (0.0)	1 (1.2)	0 (0.0)	9 (0.9)
Ancylostomatidae	1 (1.2)	2 (2.4)	5 (7.8)	12 (14.3)	0 (0.0)	0 (0.0)	1 (1.2)	6 (7.1)	4 (4.8)	12 (14.3)	9 (10.1)	32 (35.2)	14 (16.5)	98 (9.9)
*Strongyloides* *stercoralis*	0 (0.0)	0 (0.0)	0 (0.0)	0 (0.0)	0 (0.0)	1 (2.0)	0 (0.0)	0 (0.0)	0 (0.0)	0 (0.0)	0 (0.0)	0 (0.0)	0 (0.0)	1 (2.0)
*Cystoisospora* *felis*	6 (7.1)	4 (4.8)	0 (0.0)	5 (6.0)	0 (0.0)	2 (4.1)	7 (7.9)	9 (10.6)	6 (7.1)	13 (15.5)	17 (19.1)	23 (25.3)	9 (10.6)	101 (10.2)
*Cystoisospora* *rivolta*	1 (1.2)	0 (0.0)	0 (0.0)	0 (0.0)	0 (0.0)	0 (0.0)	1 (1.2)	2 (2.4)	0 (0.0)	0 (0.0)	8 (9.0)	2 (2.2)	0 (0.0)	14 (1.4)
*Giardia* *duodenalis*	0 (0.0)	0 (0.0)	0 (0.0)	2 (2.4)	0 (0.0)	0 (0.0)	2 (2.2)	0 (0.0)	0 (0.0)	0 (0.0)	3 (3.4)	4 (4.4)	0 (0.0)	11 (1.1)
*Dipylidium* *caninum*	0 (0.0)	0 (0.0)	0 (0.0)	1 (1.2)	2 (14.3)	1 (2.0)	8 (9.0)	1 (1.2)	0 (0.0)	13 (15.5)	2 (2.2)	0 (0.0)	5 (5.9)	33 (3.3)
*Taenia taeniformis*	0 (0.0)	0 (0.0)	1 (1.6)	2 (2.4)	0 (0.0)	0 (0.0)	3 (3.4)	0 (0.0)	5 (6.0)	0 (0.0)	0 (0.0)	0 (0.0)	2 (2.4)	13 (1.3)
*Mesocestoides *spp*.*	0 (0.0)	0 (0.0)	0 (0.0)	0 (0.0)	0 (0.0)	0 (0.0)	0 (0.0)	0 (0.0)	0 (0.0)	0 (0.0)	1 (1.1)	0 (0.0)	0 (0.0)	1 (0.1)
*Thelazia* spp.	0 (0.0)	0 (0.0)	0 (0.0)	0 (0.0)	0 (0.0)	1 (2.0)	1 (1.1)	0 (0.0)	0 (0.0)	1 (1.2)	1 (1.1)	0 (0.0)	0 (0.0)	4 (0.4)
Lung parasites														
Number of cats	85	84	64	84	14	49	89	85	84	84	89	91	85	987
*Aelurostrong abstrusus*	1 (1.2)	4 (4.8)	3 (4.7)	0 (0.0)	0 (0.0)	0 (0.0)	5 (5.6)	6 (7.1)	2 (2.4)	1 (1.2)	16 (18.0)	19 (20.9)	19 (22.4)	76 (7.7)
*Troglostrongylus brevior*	0 (0.0)	0 (0.0)	0 (0.0)	0 (0.0)	2 (14.3)	0 (0.0)	0 (0.0)	3 (3.5)	1 (1.2)	0 (0.0)	4 (4.5)	2 (2.2)	0 (0.0)	12 (1.2)
*Capillaria aerophila*	1 (1.2)	3 (3.6)	3 (4.7)	2 (2.4)	0 (0.0)	0 (0.0)	1 (1.1)	7 (8.2)	1 (1.2)	0 (0.0)	1 (1.1)	2 (2.2)	1 (1.2)	22 (2.2)
Ectoparasites														
Number of cats	85	84	64	84	14	49	89	85	84	84	89	91	85	987
*Ctenocephalides felis felis*	16 (18.8)	12 (14.3)	17 (26.6)	14 (16.7)	6 (42.9)	25 (51.0)	23 (25.8)	13 (15.3)	32 (38.1)	15 (17.9)	28 (31.5)	45 (49.5)	44 (51.8)	290 (29.4)
*Rhipicephalus* spp.	0 (0.0)	0 (0.0)	0 (0.0)	0 (0.0)	3 (21.4)	0 (0.0)	1 (1.1)	0 (0.0)	0 (0.0)	2 (2.4)	0 (0.0)	0 (0.0)	0 (0.0)	6 (0.18)
*Ixodes* spp.	2 (2.4)	2 (2.4)	2 (2.4)	3 (3.6)	0 (0.0)	10 (20.4)	0 (0.0)	2 (2.4)	1 (1.2)	0 (0.0)	0 (0.0)	5 (5.5)	0 (0.0)	27 (3.12)
Lice	0 (0.0)	0 (0.0)	2 (3.1)	2 (2.4)	0 (0.0)	0 (0.0)	1 (1.1)	0 (0.0)	1 (1.2)	1 (1.1)	0 (0.0)	1 (1.1)	0 (0.0)	8 (0.8)
*Otodectes cynotis*	3 (3.5)	1 (1.2)	6 (9.4)	5 (6.0)	1 (7.1)	7 (14.3)	34 (38.2)	3 (3.5)	14 (16.7)	2 (2.4)	14 (15.7)	3 (3.3)	4 (4.7)	97 (9.8)
*Notoedres cati*	0 (0.0)	0 (0.0)	0 (0.0)	0 (0.0)	0 (0.0)	0 (0.0)	1 (1.1)	0 (0.0)	0 (0.0)	1 (1.2)	2 (2.2)	0 (0.0)	1 (1.2)	5 (0.5)
*Neotrombicula* *autumnalis*	0 (0.0)	0 (0.0)	0 (0.0)	0 (0.0)	0 (0.0)	1 (2.0)	0 (0.0)	0 (0.0)	0 (0.0)	0 (0.0)	1 (1.1)	0 (0.0)	0 (0.0)	2 (0.2)
*Cheyletiella* spp.	0 (0.0)	0 (0.0)	0 (0.0)	0 (0.0)	0 (0.0)	0 (0.0)	0 (0.0)	0 (0.0)	0 (0.0)	0 (0.0)	0 (0.0)	1 (1.1)	0 (0.0)	1 (0.1)
*Demodex* spp.	0 (0.0)	0 (0.0)	0 (0.0)	0 (0.0)	0 (0.0)	0 (0.0)	0 (0.0)	0 (0.0)	0 (0.0)	0 (0.0)	0 (0.0)	0 (0.0)	0 (0.0)	0 (0.0)

Values in table are presented as the number of cats with the percentage in parentheses

*TO* Turin, *MI* Milan, *PD* Padua, *PR* Parma, *BO* Bologna, *PI* Pisa, *PG* Perugia, *TE* Teramo, *BA* Bari, *CZ* Catanzaro, *NA* Naples, *SS* Sassari, *ME* Messina

Of the 35.9% cats found to be positive for any GI or BP nematodes, 20.1% were female and 15.8% were male. Of the female cats analyzed, 40.0% were positive for endoparasite infection, while 31.7% of the male cats analyzed showed endoparasites; this difference was statistically significant (*X*^2^ = 7.37, *df* = 1, *P* = 0.007). In addition, when compared to males, females had significantly higher prevalence values for *T. cati* (28.5* vs* 22.8%; *X*^2^ = 4.23, *df* = 1, *P* = 0.04) and Ancylostomatidae (13.5* vs* 6.3%; *X*^2^ = 14.44, *df* = 1, *P* < 0.001).

Colony cats had a significantly higher prevalence of endoparasite infections (45.5%; *X*^2^ = 15.25, *df* = 2, *P* < 0.001) compared to both shelter (31.0%) and privately owned cats (32.2%). Infection with *A. abstrusus* and Ancylostomatidae was significantly higher in colony and shelter cats (15.3 and 10.3%, respectively; *X*^2^ = 13.90, *df* = 2, *P* < 0.001) compared to privately-owned cats (4.5%), while the prevalence of *T. cati* infection was comparable among the colony/shelter/privately owned cats.

Overall prevalence of endoparasite infection was significantly higher in cats aged < 1 year (49.8%; *X*^2^ = 56.95, *df* = 1, *P* < 0.001) and in cats aged between 1 and 5 years of age (36.8%; *X*^2^ = 56.95, *df* = 1, *P* < 0.001), with *T. cati* infection significantly more frequent in cats aged < 1 year (42.6%; *X*^2^ = 80.68, *df* = 1, *P* < 0.001) than in the other age groups (1–5 years: 24.6%; > 5 years: 8.2%). Infection with Ancylostomatidae was significantly more frequent in cats aged between 1 and 5 years (12.4%; *X*^2^ = 6.39, *df* = 1, *P* = 0.041) than in cats in the other age categories. Prevalence of *A. abstrusus* was significantly higher in cats aged < 1 year (9.7%) and between 1 and 5 years (8.6%; *X*^2^ = 7.58, *df* = 1, *P* = 0.023). Coccidiosis was more prevalent in younger cats (20.6%).

Prevalence of endoparasite infection was directly associated with the frequency of outdoor access. Cats with an exclusively outdoor lifestyle had significantly higher infection rates for *A. abstrusus* (14.5%; *X*^2^ = 33.01, *df* = 2, *P* < 0.001) compared to cats living predominantly outdoors or predominantly indoors (5.4 and 2.5%, respectively). Cats with a predominantly/exclusively outdoor lifestyle had significantly higher prevalence values for *T. cati* (*X*^2^ = 12.87, *df* = 2, *P* = 0.02) and Ancylostomatidae (*X*^2^ = 29.73, *df* = 2, *P* < 0.001). Cats residing in the areas of southern Italy had significantly higher prevalence values for *T. cati* (*X*^2^ = 23.84, *df* = 2, *P* < 0.001), Ancylostomatidae (*X*^2^ = 37.34, *df* = 2, *P* < 0.001) and *A. abstrusus* (*X*^2^ = 33.59, *df* = 2, *P* < 0.00).

**Fig. 2:**
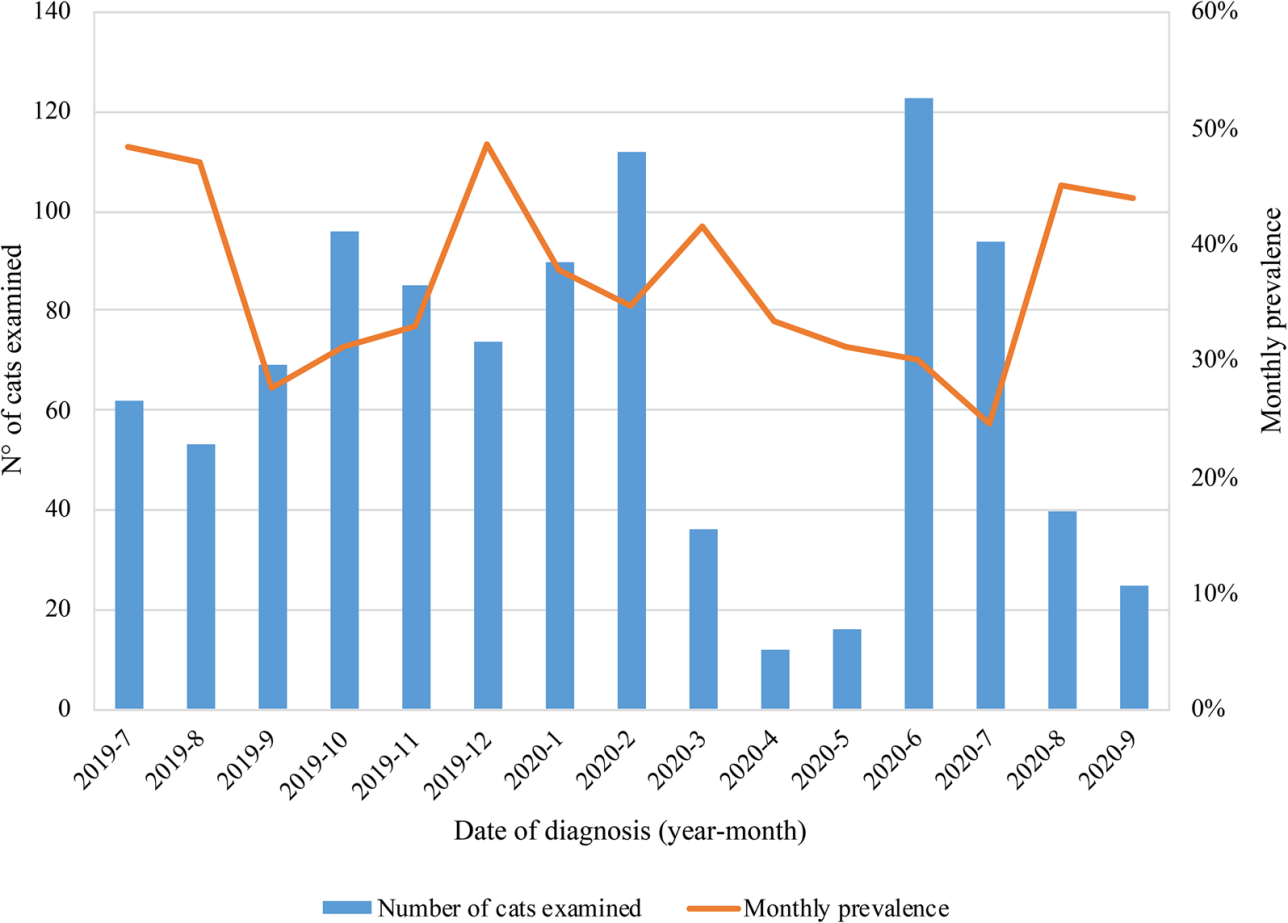
Endoparasite prevalence according to monthly evaluation

Monthly prevalence throughout the study was variable (Fig. 2), but there was no month in which parasites were not observed.

Minimum/maximum EPG/OPG/CPG results for helminths and protozoa were as follows: *T. cati* (from 5 up to 7,50,000 EPG), *T. leonina* (from 10 up to 950 EPG), Ancylostomatidae (from 5 up to 8495 EPG), *C. aerophila* (from 5 to 300 EPG), *C. felis* (from 10 up to 21,200 OPG), *C. rivolta* (from 5 up to 21,200 OPG), and *G. duodenalis* (from 10 up to 550 CPG).

### Ectoparasites

**Fig. 3:**
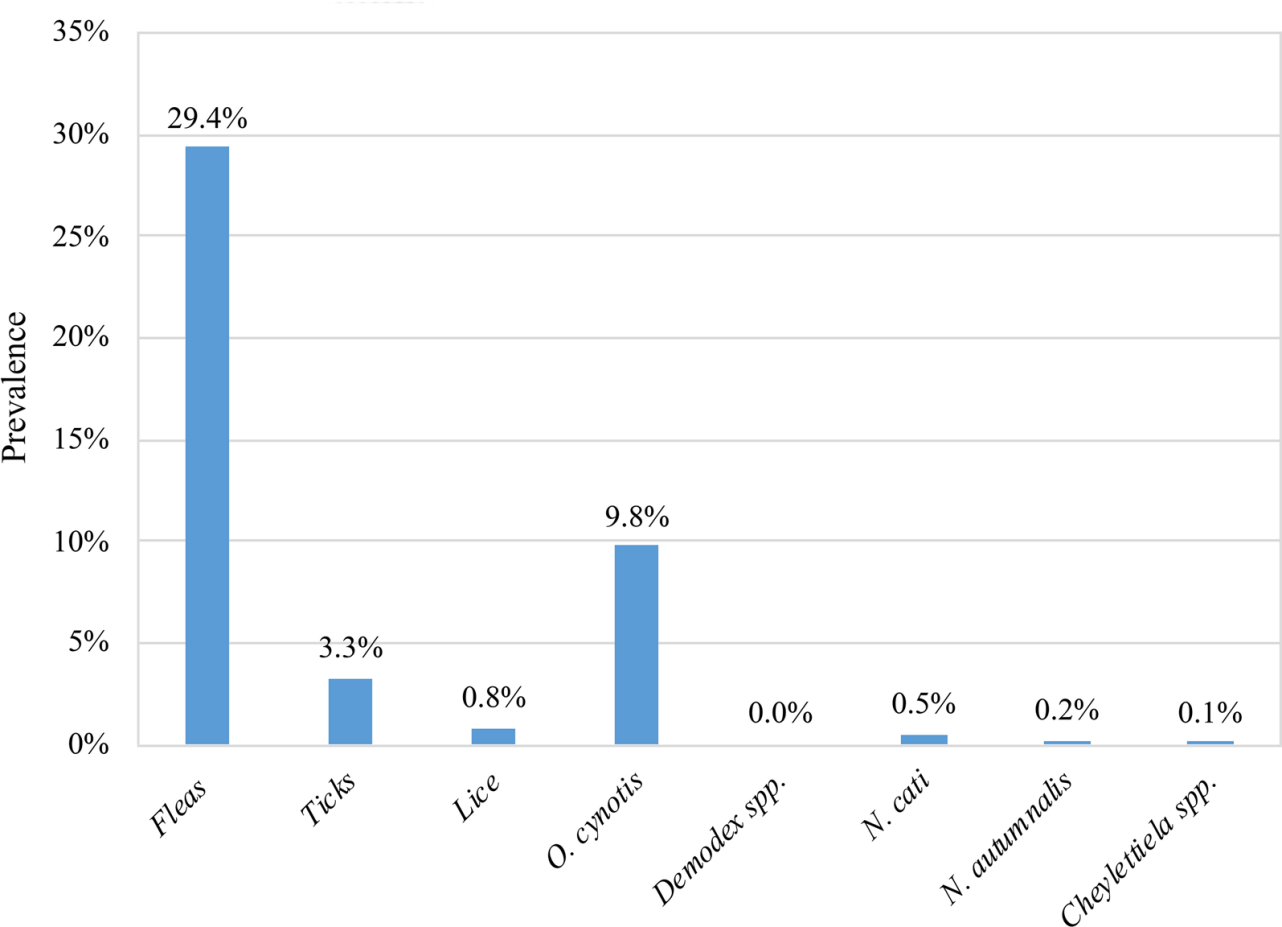
Prevalence of ectoparasite infestation

A total of 35.7% (352/987) of the study population was infested by ectoparasites. The most common parasites were fleas (*Ctenocephalides felis felis*; 29.4%, 290/987), followed by ear mites (*O. cynotis*; 9.8%, 97/987). Tick infestation (*Ixodes* spp.: 3.12%; *Rhipicephalus* spp.: 0.18%) was uncommon, as was infestation by other mites and lice [i.e. *Notoedres*: 5/987, 0.5%; *Neotrombicula*: 2/987, 0.2%; *Cheyletiella*: 1/987, 0.1%; lice (unidentified): 8/987, 0.8%] (Fig. 3; Table 2).

Of the total cat population analyzed, prevalence values for ectoparasites were comparable between male (17.3%) and female (18.3%) cats. Of the 492 females examined, 36.6% were infested, and of 495 males examined, 34.8% had ectoparasites. Overall prevalence of flea infestation for the 987 cats recruited was 14.5% in females* versus* 14.9% in males; for *O. cynotis* infestation, this was 5.7* versus* 4.2%. Considering gender specifically, 28.9 and 11.3% of examined female cats were infested with fleas and *O. cynotis*, respectively, and 29.9 and 8.3%, respectively, of male cats were infested with these two ectoparasites.

Compared to shelter and privately owned cats, colony cats had a significantly higher overall prevalence of ectoparasites (53.5%* vs* 31.0 and 28.7%, respectively; *X*^2^ = 52.65, *df* = 2, *P* < 0.001), fleas (46.5% *vs* 24.1 and 22.7%, respectively; *X*^2^ = 54.15, *df* = 2, *P* < 0.001) and ear mites (17.5%* vs* 3.4 and 7.8%, respectively; *X*^*2*^ = 25.42, *df* = 2, *P* < 0.001). The same was observed for cats with a predominantly/exclusively outdoor lifestyle [35.5 and 51.4%, respectively,* vs* 14.6% (indoor lifestyle); *X*^2^ = 81.03, *df* = 2, *P* < 0.001].

Overall prevalence for ectoparasite infestation was significantly higher in cats aged < 1 year (45.8%) and between 1 and 5 years (35.5%) (*X*^2^ = 26.25, *df* = 1, *P* < 0.001) compared to cats aged > 5 years (24.3%). Flea infestation was significantly more prevalent in cats aged < 1 year (36.5%; *X*^2^ = 15.80, *df* = 1, *P* < 0.001) and between 1 and 5 years (29.8%; *X*^2^ = 15.80, *df* = 1, *P* < 0.001) than in older cats (20.6%). The same was observed for *O. cynotis*, which showed significantly higher prevalence in cats aged < 1 year(12.3%; *X*^2^ = 7.77, *df* = 1, *P* = 0.02) and between 1 and 5 years (10.7%; *X*^2^ = 7.77, *df* = 1, *P* = 0.02) (cats > 5 years: 5.3%).

The prevalence of ectoparasites was significantly higher in cats from central (37.7%) and southern (42.7%) Italy (compared to northern area) (25.1%) (*X*^2^ = 25.97, *df* = 2, *P* < 0.001). Interestingly, the prevalence of *O. cynotis* infestation was significantly higher (19.7%; *X*^2^ = 34.80, *df* = 2, *P* < 0.001) in cats from central Italy than in those from the northern (4.8%) and southern (8.5%) areas.

**Fig. 4:**
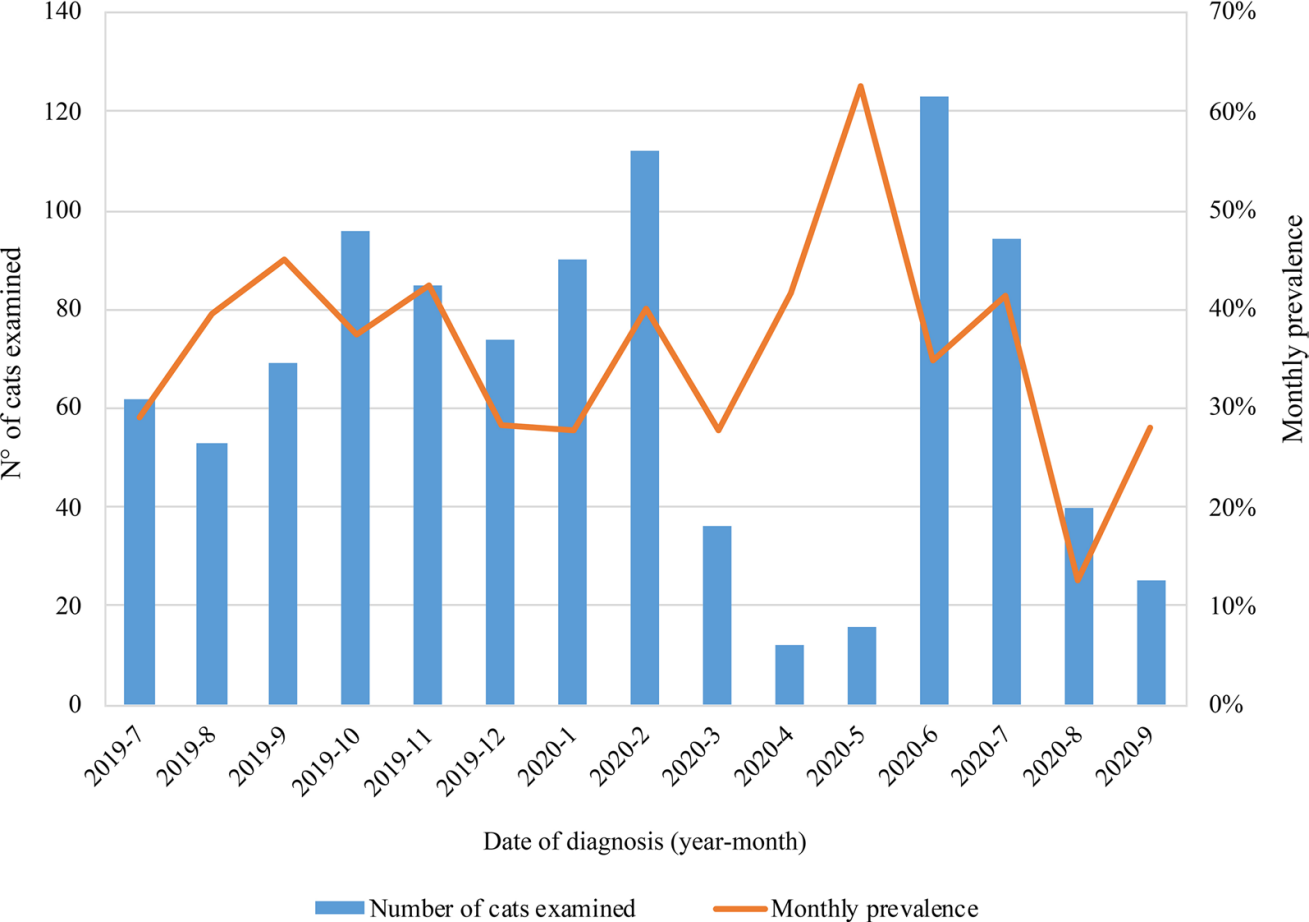
Monthly prevalence of ectoparasite infestations

Monthly prevalence throughout the study was variable (Fig. 4). Only 23.8% of the cats had received at least one treatment against endoparasites in the past year, while only 33.6% of the cats had received treatment against ectoparasites in the past year.

### Risk factors for endo–ectoparasites infestation

Significant risk factors as determined from univariate analysis were entered in the multivariable logistic regression model in order to address possible confounding factors and to compute adjusted odds ratios (OR). Tables 3 and 4 report the results of the multivariate analysis, which considered overall prevalence of endoparasite infection, overall prevalence of ectoparasite infestation and prevalence of the most common endoparasites (*T. cati*, Ancylostomatidae and *A. abstrusus*) and ectoparasites (fleas and *O. cynotis*) as dependent variables and sex, age, provenance, lifestyle, geographical area and anti-parasitic treatment as predictors. The results highlight that age was predictive for *T. cati* infection in cats aged < 1 year [OR 7.834, 95% confidence interval (CI) 4.609–13.314] and between 1–5 years (OR 3.382, 95% CI 22.021–5.661), but not for Ancylostomatidae or *A. abtrusus*. Outdoor lifestyle put cats at higher risk for all three nematodes (*T. cati*: OR 2.659, 95% CI 1.622–4.361; Ancylostomatidae: OR 3.144, 95% CI 1.285–7.691; *A. abstrusus* OR 3.558, 95% CI 1.354–9.354). Significant predictors for ectoparasite infestations included living in a colony (OR 1.612, 95% CI 1.114–2.334) and an exclusive (OR 4.497, 95% CI 2.764–7.318) or predominantly (OR 3.197 95% CI 2.084–4.905) outdoor lifestyle.

**Table 3:** Multivariable regression analyses for endoparasites

Significant predictor of risk	*P* value	OR (95% CI)
Endoparasite infection		
Female	0.047	1.331 (1.003–1.765)
Age (< 1 year)	0.000	3.953 (2.587–6.041)
Age (1–5 year)	0.000	2.198 (1.483–3.258)
Exclusively outdoor	0.000	3.515 (2.223–5.560)
South	0.000	2.052 (1.476–2.853)
No treatment	0.018	1.517 (1.074–2.143)
*Toxocara cati*		
Age (< 1 year )	0.000	7.834 (4.609–13.314)
Age (1–5 year )	0.000	3.382 (2.021–5.661)
Exclusively outdoor	0.000	2.659 (1.622–4.361)
South	0.018	1.542 (1.076–2.208)
Ancylostomatidae		
Female	0.001	2.218 (1.390–3.537)
Predominantly outdoor	0.012	3.144 (1.285–7.691)
South	0.000	3.277 (1.874–5.730)
No treatment	0.006	2.472 (1.300–4.700)
*Aelurostrongylus abstrusus*		
Exclusively outdoor	0.010	3.558 (1.354–9.354)
South	0.000	5.480 (2.498–12.024)
No treatment	0.020	2.440 (1.154- 5.160)

Multivariable regression analyses were performed using IBM SPSS Statistics for Macintosh, version 27.0

Statistical significance was set at α = 0.05

*CI* Confidence interval,* OR* odds ratio

**Table 4:** Multivariable analyses for ectoparasites

Significant predictor of risk	*P* value	OR (95% CI)
Ectoparasite infection		
Age (< 1 year )	0.000	2.290 (1.518–3.455)
Colony	0.011	1.612 (1.114–2.334)
Predominantly outdoor	0.000	3.197 (2.084–4.905)
Exclusively outdoor	0.000	4.497 (2.764–7.318)
Central	0.001	1.962 (1.320–2.916)
South	0.001	1.757 (1.256–2.458)
Fleas		
Age (< 1 year )	0.017	1.699 (1.099–2.626)
Colony	0.049	1.459 (1.001–2.125)
Predominantly outdoor	0.000	3.294 (2.024–5.361)
Exclusively outdoor	0.000	5.092 (2.974–8.718)
Central	0.030	1.606 (1.046–2.465)
South	0.000	2.069 (1.448–2.958)
No treatment	0.002	1.725 (1.215–2.449)
*Otodectes cynotis*		
Age (< 1 year)	0.017	2.377 (1.164–4.854)
Colony	0.000	3.232 (1.801–5.803)
Predominantly outdoor	0.030	2.193 (1.077–4.462)
Central	0.000	4.526 (2.652–7.724)

Multivariable regression analyses were performed using IBM SPSS Statistics for Macintosh, version 27.0

Statistical significance was set at α = 0.05

## Discussion

The study provides a overview of the endo- and ectoparasites affecting Italian cats during a 15-month study period. Despite monthly recruitment being brusquely interrupted due to the SARS-CoV-2 pandemic, preventing several centers from reaching the established number of cats per month, nearly 1000 cats were analyzed. Importantly, laboratory analyses were carried out according to standardized protocols which were followed by all centers, thus reducing the risk of variability associated with different test sensitivities/specificities. It should be noted, however, that while using one method does improve comparability, having different people use the method can influence results.

The present study also applied univariate and multivariate analyses to evaluate overall risk for endoparasites or ectoparasites and for the most common helminths and arthropods observed.

Our findings show that feline endo- and ectoparasites are widespread in Italy, with varying prevalence across the different regions. Approximately 55.0% (539/987) of cats enrolled in our study were infected with at least one internal or external parasite. This compares well with the results of a study at the European level [1] which reported that more than half (50.7%) of the cats studied were infected with one or more endo- and/or ectoparasite.

Among the GI helminths, *T. cati* was the most frequently found (25.6% of enrolled cats). This prevalence is higher than that reported by a previous Italian multicenter study [4] (21.6%), and is also higher than the mean European prevalence value reported in 2017 by Giannelli et al. [2] (14.5%). The prevalence is, however, in line with that of other surveys conducted in Italy [5, 6]. In the present study, risk for *T. cati* infection included age (< 5 years of age), an exclusively outdoor lifestyle and living in southern Italy. Pre- and perinatal transmission and age immunity are well known for *Toxocara* spp., while outdoor access favors exposure to both highly resistant eggs contaminating the environment and to paratenic hosts [20]. The warmer climatic conditions in southern Italy and Italian islands likely increase the presence and persistence of infective stages of the parasite [21].

Ancylostomatidae represented the second most frequent group of nematodes diagnosed in the study population (9.9%). In a study performed at the European level [2], the mean prevalence of hookworms was reported to be 4.5%, while in some selected areas of Italy the prevalence was reported to be 4.9% [4]. In the present study, Ancylostomatidae infection was associated with gender (females were at increased risk), predominantly outdoor access, living in southern Italy and lack of anthelmintic treatment. Age was not a significant risk predictor. Indeed, it is assumed that there is no transmammary transmission of Ancylostomatidae from the queen to her kittens [22].

As mentioned above, warmer climatic conditions in southern Italy may increase the presence and persistence of infective stages of Ancylostomatidae. For transmission to occur, Ancylostomatidae need to develop into infective larvae in the soil from eggs passed in the host’s stool, and higher temperature and humidity (tropical and subtropical climates) provide an adequate environment for this growth stage [23].

The feline lungworm *A. abstrusus* was present in 7.7% of the cats analyzed in our study; in comparison, Giannelli et al. [2] reported a mean prevalence in Europe of 8.2%, while recent data from Italy [4] reported a prevalence of 10.3%. Multivariate analysis indicated that an exclusively outdoor lifestyle, living in southern Italy and lack of anthelmintic treatment were significant risk factors for *A. abstrusus*. Outdoor access has been reported previously as an important risk factor for *A. abstrusus* [24]. While the geographical distribution of feline lungworms tends to be patchy but stable in endemic hotspots [2], interpretation of geographical location as a risk factor should be done with caution, and any geographic location reporting autochthonous circulation of the parasite should be considered to be potentially endemic. Traversa et al. [4] reported a 20.0% prevalence of *A. abstrusus* in Piedmont, while in the present study only 1.2% of cats were infected. Giannelli et al. [2] reported that 16.7% of cats from the province of Bari were infected with *A. abstrusus*, compared to a prevalence of 2.4% in our study. Values from the provinces of Sassari (SS) and Messina (ME), on the other hand, were higher in the present study (20.9 and 22.4%, respectively) (Table 2), compared to data reported by Giannelli et al. [2] (11.6 and 15.3%, respectively), but lower than the prevalence of 25.2% reported by Tamponi et al. [25] in a previous study in Sardinia. Interestingly, univariate analysis showed that cats aged > 5 years had a significantly higher prevalence for *A. abstrusus*, but age was not confirmed as a risk factor following multivariate evaluation. As expected, larvae of *T. brevior* were found mostly in cats from southern regions. Nevertheless, it is worth mentioning that, in the present study, this potentially fatal metastrongyloid was also found in two cats in northern Italy, indicating an apparent northward geographical expansion.

The Mini-FLOTAC technique [7] has been recently demonstrated to be a highly sensitive method for diagnosing parasitic infections of human and veterinary importance where larvae or ova of parasites are shed in the feces [8–11]. Moreover, the mini-FLOTAC technique in combination with Fill-FLOTAC has been shown to be user-friendly and safe, with a wide diagnostic range. These features are particularly useful for monitoring and control programs in which large numbers of fecal samples need to be processed rapidly and safely. In the present study, the harmonized use of the Mini-FLOTAC technique allowed the qualitative and quantitative analysis of parasite load without the need for specialized equipment.

Fleas were the most common ectoparasite found in the present study (29.4%). Cooper et al. [26] reported similar prevalence values in a recent nationwide study in the UK. Beugnet et al. [1] reported prevalence values ranging from 3.6% in Bari to 31.4% in Naples. Multivariate analysis showed that infestation was associated with young age (< 1 year), living in a colony, predominantly/exclusively outdoor lifestyle, living in central and southern Italy and lack of ectoparasitic treatment. Cooper et al. [27] reported geographical differences (prevalence declining from south to north in UK) and no treatment as significant predictors of risk for flea infestation. Beugnet et al. [1] identified outdoor access as the only risk factor in a multivariate analysis.

The ear mite *O. cynotis* was present in 9.8% of enrolled cats. Beugnet et al. [1] reported prevalence values of 40.3% in cats from Bari and 21.8% in cats from the area of Naples. In Tuscany (central Italy), *O. cynotis* was identified in 66.1% cats with otitis externa [27]. Age < 1 year, living in a colony, having a predominantly outdoor lifestyle and coming from central Italy were all factors identified as increasing the risk of ear mite infestation in cats. *Otodectes cynotis* is transmitted by direct contact and is highly contagious. Young cats are more playful and likely have more direct contact with other cats.

The main limitation of the present study is the potential effect of bias, based on inclusion criteria. While on the one hand cats that had received antiparasitic treatment in the 3 months preceding enrollment were excluded, the evaluation of the effect of treatment* versus* no treatment was carried out considering the frequency of treatment for the 12 months prior to enrollment (see Additional file 1: Text S1). Beugnet et al. had a similar study design [1], including cats that had received no treatment in the 2 months prior to enrollment and analyzing the frequency of antiparasitic treatment as a risk factor for infection. Outdoor access was another enrollment bias. Indeed, this has already been identified as a risk factor for parasite infection in cats [1–3]. In the present study, however, access was categorized as infrequent, frequent or exclusive. The analysis of risk was based on the frequency of access, not on outdoor* versus* indoor.

## Conclusions

The results of this study highlight the fact that both ecto- and endoparasites are still common in cats throughout Italy. Interestingly, of the 239 cats with a predominantly indoor lifestyle, 31.8% were affected by endo- and ectoparasites, suggesting that parasiticide treatment is more important than lifestyle. Therefore, taking the zoonotic implications and the clinical importance into account, it is strongly advisable to promote effective and regular parasite control in cats, with adequate frequencies of treatment for both internal and external parasites. It is interesting to note that there was no month in which endo- and ectoparasites could not be found, suggesting that cats can be infected throughout the year. This would imply that parasite infection should not be considered seasonal, but that control should be year-round. However, only 23.8% of the cats had received at least one treatment against endoparasites in the past year, while only 33.6% of the cats had received treatment against ectoparasites in the past year. The European Council for the Control of Companion Animal Parasites (ESCCAP) recommends “year-round, life-long” parasite control. The aim of the present study was not to associate climate and environmental conditions to risk of parasite infection, but rather to support these recommendations. Furthermore, many privately owned cats spend a significant amount of time outdoors where they are exposed to parasites. Practitioners need to inform their clients of the risks and recommend *routine* antiparasitic treatment. Interestingly, southern Italy continues to show higher prevalence of parasite infection in cats. This may be due to climatic, social or economic factors, and practitioners working in these areas should be particularly attentive.

Finally, zoonotic parasites and vectors of human disease are still widespread in cats, confirming the need to protect the human–animal bond and the application of the One-Health concept.

## Supplementary Information

**Additional file 1**: **Text S1.** Study Form 2 Enrollment.

## Data Availability

All data generated or analyzed during this study are included in this published article.

## Abbreviations

BP: Broncho–pulmonary
GI: Gastro–intestinal nematodes
