# Supplementary material for: Prevalence and risk factors associated with cat parasites in Italy: a multicenter study
Source: Parasit Vectors. 2021 Sep 15;14:475. doi: 10.1186/s13071-021-04981-2 (PMC8441231; doi:10.1186/s13071-021-04981-2)
Supplement: Supplementary file 1 — Additional file 1: Text S1. Study Form 2 Enrollment. [file 13071_2021_4981_MOESM1_ESM.docx]

**Inclusion criteria**:

Endo-ectoparasiticide treatment in the last three months **□** **No □** Yes**^(1)^**

*(1) if “yes” the cat cannot be included in the study.*

Does the cat have access to the outdoors? **□** **Yes** **□** No**^(1)^**

*(1) if “no” the cat cannot be included in the study.*

**Name of cat**: ________________ **Breed**:________________ **Date of enrolment**: _ _ / _ _ / _ _

**Address,** via:_________________ comune: ________________ provincia:_________ CAP: ________

**Age**:_____ **□ Wks**. **□ Ms.** **□ Years** **Sex**: **□** M **□** F Neutered/Spayed: **□** **Yes** **□** No

**Provenance:** shelter □, colony □, owned: **□**, *other ________________________*

**Lifestyle**: □ predominantly indoor, □ predominantly outdoor, □ exclusively outdoor

**Other animals**: Cats: **□** Yes **□** No n.___**,** Dogs: **□** Yes **□** No n.___**,** Other **□** Yes **□** No, Specify ___________

**Clinical observations**

| **Nutritional state**: | | | **□** normal | | | **□** abnormal | | ________________________________ | |
| --- | --- | --- | --- | --- | --- | --- | --- | --- | --- |
| **Gastro-intestinal tract**: | | | **□** normal | | | **□** abnormal | | ________________________________ | |
| **Cardio-resp**: | | | **□** normal | | | **□** abnormal | | ________________________________ | |
| **Skin/haircoat**: | | | **□** normal | | | **□** abnormal | | ________________________________ | |
|  | | |  | | |  | |  | |
| **Presence of ectoparasites?:** | | | fleas: | | **□** Yes **□** No | | (*collected:* **□** Yes **□** No) | | |
| ticks: | | | | | **□** Yes**□** No | | (*collected:* **□** Yes **□** No) | | |
| lice: | | | | | **□** Yes **□** No | | (*collected:* **□** Yes **□** No) | | |
| *Otodectes cynotis*: | | | | | **□** Yes **□** No | | *Notoedres cati*: | | **□** Yes **□** No |
| *Demodex* spp.: | | | | | **□** Yes **□** No | | *Trombicula autumnalis*: | | **□** Yes **□** No |
| *Cheyleitiella* spp.: | | | | | **□** Yes **□** No | |  | | |
| other: | | | | | **□** Yes **□** No | | _____________________________________ | | |
| **Eyes**: | **□** normal | **□**abnormal | | __________________________________________________ | | | | | |
| *Thelazia callipaeda:* | | **□** Yes **□** No | | | | |  | | |
| **Other**: | **□** normale | **□** anormale __________________________________________________ | | | | | | | |
| **Last anti-parasitic treatment**: _______________________ **Date**: ______ | | | | | | | | | |
| *Commercial name* | | | | | | | | | |
| **Number of treatments in the last year**: insecticide/acaricide: ____ endoparasiticide: ____ | | | | | | | | | |
| **Samples**: **Feaces** (7 g) **□** Yes **□** No  **min. flea comb** **□** Yes **□** No | | | | | | | | | |
| Comments:_________________________________________________________________________ | | | | | | | | | |
|  | | | | | | | | | |
|  | | | | | | | | | |

Signed: ________________________
